# SAM-Support-Based Electrochemical Sensor for Aβ Biomarker Detection of Alzheimer’s Disease

**DOI:** 10.3390/bios13080809

**Published:** 2023-08-11

**Authors:** Phan Gia Le, Hien T. Ngoc Le, Hee-Eun Kim, Sungbo Cho

**Affiliations:** 1 Department of Electronic Engineering, Gachon University, Seongnam-si 13120, Republic of Korea; legiaphan2020@gachon.ac.kr (P.G.L.); ngochien1809@gmail.com (H.T.N.L.); 2 Department of Dental Hygiene, Gachon University, Incheon 21936, Republic of Korea; hekim@gachon.ac.kr; 3 Department of Health Sciences and Technology (GAIHST), Gachon University, Incheon 21999, Republic of Korea

**Keywords:** Alzheimer’s disease, electrochemical detection, self-assembled monolayer, nanomaterials

## Abstract

Alzheimer’s disease has taken the spotlight as a neurodegenerative disease which has caused crucial issues to both society and the economy. Specifically, aging populations in developed countries face an increasingly serious problem due to the increasing budget for patient care and an inadequate labor force, and therefore a solution is urgently needed. Recently, diverse techniques for the detection of Alzheimer’s biomarkers have been researched and developed to support early diagnosis and treatment. Among them, electrochemical biosensors and electrode modification proved their effectiveness in the detection of the Aβ biomarker at appropriately low concentrations for practice and point-of-care application. This review discusses the production and detection ability of amyloid beta, an Alzheimer’s biomarker, by electrochemical biosensors with SAM support for antibody conjugation. In addition, future perspectives on SAM for the improvement of electrochemical biosensors are also proposed and discussed.

## 1. Introduction

Nowadays, Alzheimer’s disease (AD) is receiving increasing attention as its burden on society due to memory and recognition impairment relating to neurodegeneration [1]. Based on statistical data, in 2020, the number of Alzheimer patients was estimated to be around 50 million and is predicted to be roughly 152 million by 2050 [2]. The expenditure for serving AD patients is about USD one trillion annually [2]. Thus, reducing the number of patients and the economic burden is extremely necessary.

**Figure 1:**
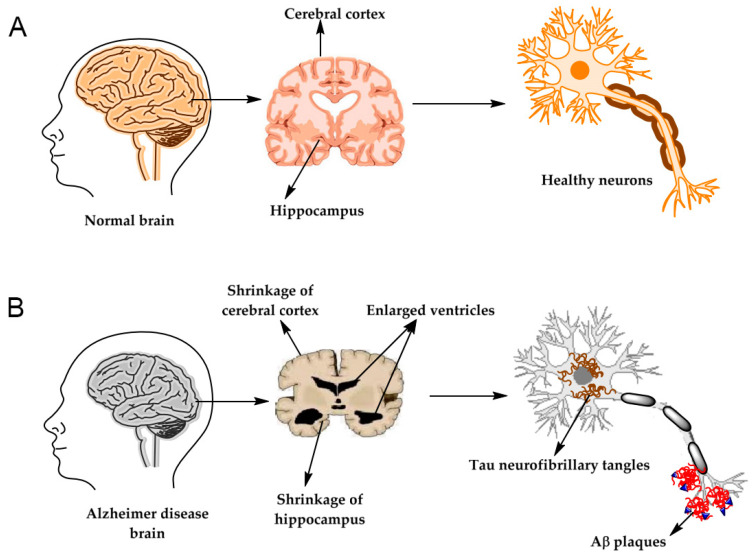
Illustration of the brain and neurons structures for both cases of (**A**) brain of a healthy person and (**B**) brain of a person with Alzheimer’s [2].

**Figure 2:**
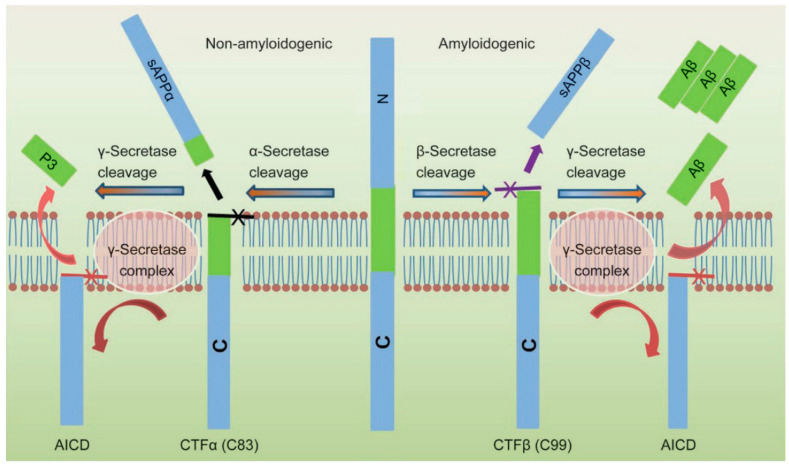
Mechanism for the Aβ peptides formation from human APP proteolysis [10].

AD, like Parkinson’s disease, is linked to neurodegeneration [3,4]. The illustration of brain images observed from Alzheimer’s patients is described in Figure 1. There are many hypotheses about the formation of this disease; the appearance of the amyloid-beta oligomer (AβO) tangle and fibrils in the brain derived from Aβ aggregation has been widely accepted by numerous researchers, as documented by high Aβ concentrations in human blood and cerebrospinal fluid (CSF) obtained from clinical research [1]. Thus, the biomarker of Amyloid beta protein (Aβ_1-40_ and Aβ_1-42_), phosphorylated tau (p-tau) aggregation, causes neurotoxicity [4,5]. The production of Aβ originates from the cleavage of the amyloid precursor protein (APP) by β- and γ-secretase, whereas cleavage by α-secretase is non-amyloidogenic [1]. The progress of Aβ formation occurs in two steps: first, β-secretase cleaves APP to form C99; next, γ-secretase continues cleaving C99 to generate Aβ of a different length, consisting mainly of Aβ_40_ and Aβ_42_, as presented in Figure 2. An insignificant number of researchers have studied acetylcholine esterase, acetyl choline, and choline as biomarkers associated with AD [2,6,7,8,9]. Biosensors capable of detecting these biomarkers for diagnosis and early warning to support AD treatment have been intensively researched currently. Thus, circumventing maintaining challenges in AβO-based biosensors will be crucial in enabling the diagnosis and treatment of AD.

Research on the detection of AβO has taken diverse approaches, such as conventional [11,12,13,14], optical [15,16,17,18], electrochemical [19,20,21], and electroluminescent methods [22,23,24,25], and quartz crystal microbalance [26,27,28]. Among these, conventional detection methods utilize magnetic resonance imaging (MRI) [13,14,29], near-infrared fluorescent (NIRF) [16,30,31], and positron emission tomography (PET) [11,12,32,33], which are time-consuming, have low spatial resolution [34], and are also expensive as well as cause side effects such as vomiting, flushing, itching, headache, and nausea [4]. In contrast, the colorimetric method has the pros of visualization, a low cost, and easy operation, but the high limit of detection (LOD) makes it not useful enough for Aβ biomarker detection [35,36,37]. However, the fluorescent method sometimes enables improvement in the LOD at the picomolar or femtomolar level [38,39]. Electrochemical sensors are well-known for their quick reaction rate, high precision, high sensitivity, good controllability, and instantaneous responsibility [40]. Moreover, the fabrication of electrochemical sensors is straightforward, pre-treatment of a clinical sample is not required, and sample volume is insignificant [41]. In addition, it has a wide range of applications, namely clinical diagnosis, biomedical research, food quality management, and environmental monitoring [42]. Especially, electrochemical sensors are compatible with miniaturized devices, which are appropriate for detecting biomarkers with an ultra-low concentration in human serum [43,44], saliva [45,46], blood [47,48], CSF [49,50], and plasma [51,52,53]. The operation of Aβ-based electrochemical sensors related to AD has been reported by a significant number of scientists obtaining high sensitivity and selectivity.

A self-assembled monolayer (SAM) has been applied in chemical sensors, biosensors, batteries, and electronic devices [54,55,56]. In electrochemical sensors, SAM is presented as a conjunction layer on the working electrode surface, which is subjected to electrode modification towards improvement in sensor selectivity and sensitivity relying on functionalities for immobilizing and orientating enzymes and antibodies [4,34,57,58,59]. So, an SAM-appropriate design and fabrication can enhance electrochemical sensor performance. In addition, reported SAM-based electrochemical sensors for Alzheimer’s biomarker detection obtained an ultralow detection limit, which reflects that the utilization of SAM is suitable in the development of such electrochemical sensors. To the best of our knowledge, no publication has discussed the SAM of modified electrodes for Aβ peptide detection. Therefore, the current review presents and discusses SAM-related electrochemical sensors. The trends and challenges of electrochemical sensors are also discussed.

## 2. Overview of Electrochemical Sensors

### 2.1. Electrochemical Sensor Architecture

An electrochemical sensor is an electrical equipment module which receives electrical signals derived from biochemical reactions taking place between molecules, which is a redox progression [4,60]. Basically, electrochemical sensor architecture consists of three electrodes: the working electrode, reference electrode, and counter electrode [40]. Among these, the working electrode is a crucial component, as the main reaction occurs on its surface, and thus it determines the sensitivity, selectivity, and durability of the sensor. Many kinds of working electrodes have been developed to enhance the selectivity and sensitivity of Aβ electrochemical sensors; these include glassy carbon electrode (GCE) [53,61], paper-based carbon electrode [62,63], screen-printed electrode (SPE) [64], Ni-foam electrode [65], and interdigitated microelectrode [4,34,57,66,67]. Amongst these, GCE is very popular but not suitable for miniaturized devices, paper-based carbon electrode is inexpensive and disposable, screen-printed and Ni-foam electrodes are easily fabricated, and interdigitated microelectrode is highly sensitive but requires a prohibited machine and trained technician. Pristine working electrodes for electrochemical sensors with intrinsic physiochemical properties hinder the detection of the Aβ biomarker, which can be improved by tailoring one or many layers of materials on the surface, such as novel metal [62,63], carbon [61,64], composite [68,69], or a conductive polymer [70] to boost the sensor’s sensitivity. Generally, electrochemical electrodes are expensive, whereas they possess higher sensitivity than other reported method counterparts [69,71], which meets the requirements for biomedical application. In addition, the expenditure of electrochemical sensor production can be reduced by scaling up; specifically, paper-based screen-printed electrodes are thin, easy to fabricate, not time-consuming, easily mass-produced, highly economical, and disposable, which will be crucial to point-of-care systems in the future. Hence, suitably modified electrodes can improve the detection of the Aβ biomarker down to an ultra-low level in human serum, saliva, CSF, and plasma.

### 2.2. Operational Principle

Electrochemical sensor operation is based on the redox reaction of the analyte on the working electrode surface accompanied by electrical signal generation, which varies with different analyte concentrations [72,73,74]. The signal is received by the detector of the analyzer, e.g., voltammetry analyzer, impedance analyzer, and so on. Changes in the analyte concentration usually induce a change in the responsive electrical current, allowing for the establishment of a calibration plot between the target concentration and signal intensity [20,53,63], from which the analyte concentration can be determined by extrapolation by applying a mathematical algorithm. Based on the manner that the signal was collected in, diverse techniques such as cyclic voltammetry (CV) [42,75], amperometry [73,76], electrochemical impedance spectroscopy (EIS) [77,78], differential pulse voltammetry (DPV) [74,79], and square wave voltammetry (SWV) [80,81] were used. Amongst these, DPV, EIS, and SWV are considered to be better at improving sensitivity compared with CV and are widely applied for Aβ detection, which will be discussed in the next section.

## 3. Recent Research on Aβ Detection by Electrochemical Sensors

### 3.1. Electrochemical Sensors for Aβ Detection

Many studies have demonstrated that Aβ can be completely detected by employing an electrochemical sensor to obtain a low LOD at picograms or femtograms per milliliter [53,63,64,67,69,70,71,82]. The Aβ peptide structure has a tyrosine group, histidine, and methionine which participate in the redox reaction with an oxidized peak at ~0.6 and ~1.05 and wave at 1–1.5 V vs. Ag/AgCl on a carbon surface [83,84]. The oxidation occurring on the residual groups under a supplied voltage indicates the detection ability for Aβ biomarkers; however, this method detects a plethora of other proteins containing such groups as well. Non-specific adsorption during the detection of such proteins could be eliminated by designing an electrochemical immunosensor in which non-specific adsorption is prevented by blocking the residual surface with bovine serum albumin (BSA) [34,53,69].

The anti-Aβ antibody and Aβ antigen interaction taking place on the working electrode surface can be detected by diverse techniques such as CV [42], amperometry [73], DPV [63,64,69,70,72,85], EIS [53,62,65,71], and linear square voltammetry (LSV) [75]. Depending on the requirements, available equipment, and labor ability, each technique can be applied reasonably and effectively. Moreover, Aβ peptides can be detected using labels with enzymes, nanozymes, or anti-IgG-ALP, [86,87] or label-free using functional groups [88,89,90] in electrochemical detection methods.

### 3.2. Working Electrode Modification

**Figure 3:**
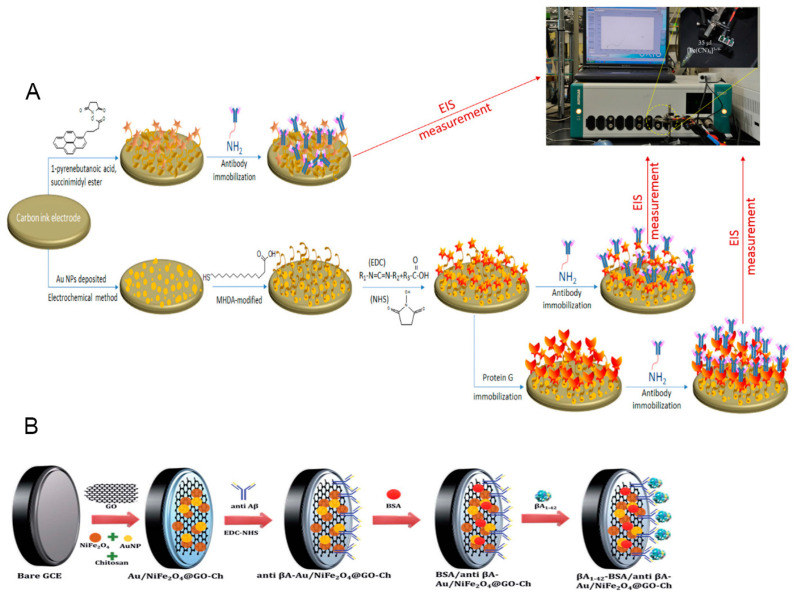
Tailoring the working electrode for the electrochemical biosensor. (**A**) The Au-based electrode was modified with an SAM, then orientation was assisted with protein G, and, finally, conjugated antibody Aβ [55]. (**B**) The bare GCE electrode was modified with Au and composite of NiFe_2_O_4_@GO-Ch, then we conjugated the antibody [65]. SAM, self-assembled monolayer.

The use of bare electrodes has shortcomings, such as low sensitivity, selectivity, and durability, which hamper the applicability of the Aβ electrochemical sensor due to a lack of an appropriate detection range and an inappropriate LOD, leading to it being less reliable and limiting the diagnostic application. To overcome these bottlenecks, many methods have been employed to modify the electrode surface, including designing different kinds of electrodes, synthesizing diversely structured nanomaterials, or amplifying the detectable signal. Among these, designing different kinds of electrodes requires expensive equipment along with skilled labor and amplifying the detectable signal is limited by a physical threshold, whereas diverse nanostructured materials on working electrodes have been intensively applied in laboratory environments employing metal, metal oxide, carbon, composite, conductive polymer, and SAM. Practically, tailoring the materials on the electrode surface to achieve a better performance has been conducted in a variety of research areas, like energy [91,92], electrochemical sensors [61,63,93], etc. Electrode-modified electrochemical sensors prove their effectiveness by obtaining high sensitivity and selectivity [61,63,93], as illustrated in Figure 3. In this study, SAM-related content is focused on; however, to have an overview and enhance the logical comprehension of the present review, electrode modification based on the remaining kinds of materials is also discussed and outlined.

#### 3.2.1. Metal, Alloy, and Metal Oxide

The noble metal Au has been documented to enhance the Aβ peptide detective performance of electrochemical sensors. Dai et al. deposited a thin film of Au on the working and counter electrodes by vapor deposition on the atomic level [85]. The SAM used 3-Mercaptopropionic acid (MPA), then EDC and NHS were added for functionalization to conjugate antibody Aβ42. Aβ42 was detected using the DPV technique with 5 mM [Fe(CN)_6_]^3−/4−^ at various incubation times. The experiments were conducted in undiluted human serum at a linear range of 67.5–500 ng/mL. Lien et al. prepared a disposable electrochemical-printed (DEP) chip with Au deposition using CV techniques [62]. Then, antibodies were conjugated on the SAM layer to detect the Aβ peptides using the electrochemical impedance technique with 1–10^3^ nM and an LOD of 2.65 nM. Further modification of the electrode with G protein increased the ability to detect Aβ(1-42) with a linear range of 10 pM–100 nM and an LOD of 0.57 nM. Xia et al. reported an electrochemical immunosensor for the detection of Aβ oligomers (AβOs) [82], in which PrP(95-110) was labeled with adamantine (Ad) to form Ad-PrP(95-110), and then Ag nanoparticles (NPs) were add to a Ad-PrP(95-110) solution to form a mixture that was anchored onto a β-cyclodextrin (β-CD)-covered electrode surface through host–guest interaction; finally, the cyclodextrin-covered electrodes were incubated overnight with β-CD-SH and TCEP to link with the plate gold electrode, and the unreacted gold surface was blocked with the MCH solution. The fabricated sensor detected AβOs with a linear range of 20–100 nM and an LOD of 8 pM.

In addition to pure metal, alloy has also been used in electrochemical biosensors for detecting Aβ, as reported by Liu et al. [63]. They prepared an electrochemical aptasensor based on a combination of AuPt alloy nanoparticles and carbon fiber paper (CFP) to form CFP/AuPt which was then incubated with DNA aptamer to detect Aβ oligomers with a linear range of 0.5–10^4^ pg/mL and an LOD of 0.16 pg/mL.

In addition, metal oxide has also been used for Aβ electrochemical sensors. Supraja et al. designed an electrochemical immunosensor relying on SnO_2_ nanofibers (SNFs) [49]. SnO_2_ metal oxide was drop-cast on the GCE surface and then functioned with -COOH to conjugate anti-AB42-antibody for immunoreaction. The fabricated sensor was able to detect β-amyloid(1-42) (AB42) with a linear range of 1 fg/mL to 10 ng/mL and an LOD of 0.146 fg/mL for AB42 spiked buffer, and linear range of 1 fg/mL to 1 ng/mL and an LOD of 0.638 fg/mL for plasma samples. The sensor can survive for over 126 days.

Metal oxide is inexpensive compared with novel metal and alloy, but it has proven to be effective in Aβ detection. Thus, the applicability of such materials in this kind of biosensor is immense.

#### 3.2.2. Carbon-Based Materials

Carbon materials are famous for their high surface area, conductivity, and flexibility, which acts as a scaffold for tailoring or anchoring other elements in electrochemical biosensor applications. In addition, it contains many functional groups such as hydroxyl (-OH) and carboxyl (-COOH) which can be functionalized to conjugate antibodies for the antigen detection of Aβ. Sethi et al. designed a label-free electrochemical sensor based on the dual layer of graphene oxide and reduced graphene oxide to detect plasma-based Aβ_1-42_ [64], which was modified with 1-pyrenebutyric acid N-hydroxysuccinimide ester (Pyr-NHS) to assist in H31L21 antibody immobilization. As a result, Aβ_1-40_ was recognized with a linear range of 11–55 pM and an LOD of 2.398 pM. Chae et al. also constructed a carbon-based electrochemical sensor with the enhanced surface functionality of reduced graphene oxide (rGO) for diagnosing Alzheimer’s disease [67]. GO was deposited on the substrate in 20 layers and was then treated with hydriodic acid (HI) to form a thin film of rGO, which was further treated to create a photo-resistant pattern, and finally lifted off; photolithography was then used to form the gold electrodes. The electrode was then incubated with EDC/NHS to immobilize 6E10 monoclonal antibody via covalent bonds with ethanolamine (ETA) to avoid any undesired covalent bonds. The oxygen-plasma-treated electrode exhibited a response 1.68-fold superior to that of the untreated electrodes in Aβ_42_ detection. Ji et al. investigated the effect of various materials functionalized on graphene for Aβ detection [94] and recognized that the presence of Aβ on the surface of materials strongly affected electron transport. Hence, carbon-based materials are advantageous for fabricating electrochemical sensors for Aβ peptide detection.

#### 3.2.3. Composite Materials

Composite materials are widely utilized for electrochemical sensors in general and for electrochemical biosensors in particular thanks to the synergetic effect between diverse materials to enhance the electrical signal and, therefore, improvement in the sensitivity and stability. Zhou et al. prepared an electrochemical aptasensor of Au-deposited vertical graphene/carbon cloth (VG/CC), and then cellular prion protein (PrPC) residues 95-110 were immobilized on the electrode surface based on the Au-S bond to Aβ oligomer [69]. Finally, poly(themine)-template Cu NPs were used as electrochemical probes for the aptasensor. The fabricated aptasensor detected Aβ with a linear range of 10–2200 pM and an LOD of 3.5 pM. In another report, Li et al. prepared bifunctional Pd-decorated Co_9_S_8_ polysulfide nanoparticles supported on graphene oxide (G/Co_9_S_8_-Pd) [71], which was deposited on the surface of GCE as a substrate for antibody conjugation by linking with Pd NPs after 1 h of incubation. The label-free electro immunosensor detected Aβ peptides with a linear range of 0.1 pg/mL–50 ng/mL and a low LOD of 41.4 pg/mL. Composite materials are promising candidates for electrochemical biosensor applications by improving the LOD toward higher sensitivity, which is a desired property for the design and practical application of biosensors.

#### 3.2.4. Conductive Polymer

Conductive polymer with high electrical conductivity is also used for electrochemical sensors. In addition, functional groups on the structural surface facilitate the conjugation of antibodies for immunoreaction. Abbasi et al. prepared an electrochemical sensor with a conductive polymer with a controlled thickness on the screen-printed electrode [70]. Ultra-thin layers of polymerized 1,5-diaminonaphthalene (pDAN) were coated on the graphene layer of the electrode at a controlled thickness, and the anti-beta amyloid antibody was activated in a solution of EDC/NHS and conjugated on the conductive polymer; the free amine group was blocked by BSA. The sensor could detect Aβ_42_ with a linear range of 1–1000 pg/mL, an LOD of 1.4 pg/mL, and a limit of quantification (LOQ) of 4.25 pg/mL. Zhao et al. prepared an electrochemical sensor with Au and PrP^c^ embedded in the conductive polymer matrix of poly(thiophene-3-acetic acid), poly(pyrrole-2-carboxylic acid), and poly (pyro-3-carboxylic acid) for the detection of AβO with a linear range of 10^−9^–10^3^ nM [95]. The PrP^c^/AuNPs-E-Ppy-3-COOH-based sensor detected Aβ with an LOD of 10^−9^ nM. Conductive polymers with the advantageous features of high conductivity, flexibility, and structure-based functional groups are useful in electrochemical sensor applications.

#### 3.2.5. SAM-Support-Based Working Electrodes for Aβ Electrochemical Sensors

**Figure 4:**
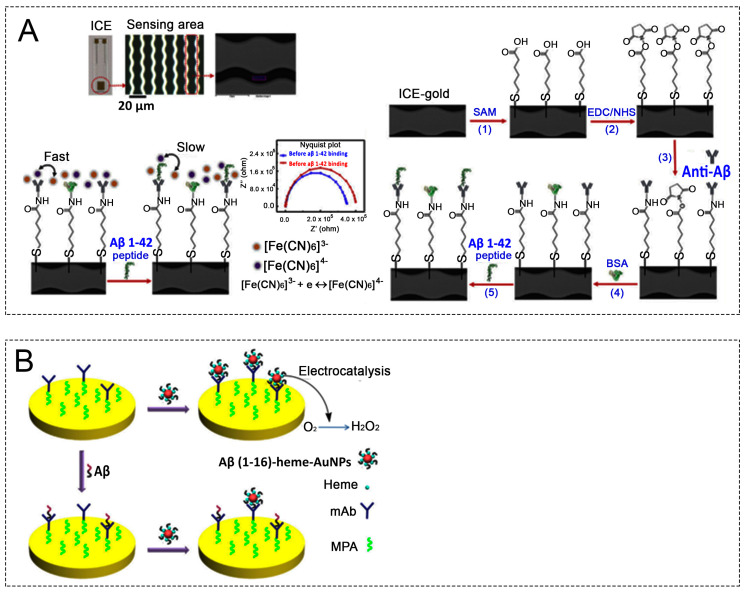
(**A**) Microscopic image of the sensing figures in ICE: Schematic of the biosensor construction at each stage of the process. The faradaic impedimetric surface engineering is based on the electron transfer resistance of the redox probe [Fe(CN)6]^3−/4−^ in solution [4]. (**B**) Schematic representation of Aβ detection: Aβ(1-16) heme-Au NPs are attached onto the mAb-covered electrode without the Aβ capture step (**top**). A smaller number of Aβ(1-16)-heme-Au NPs are attached after incubation of the electrode with Aβ species (**bottom**) [38]. ICE, interdigitated chain-shaped electrode; NPs, nanoparticles.

Self-assembled monolayer (SAM) is ordered arrays of organic molecules and has been employed as monolayers on electrode surfaces in liquid or solid phases in many electronic devices, such as electrochemical sensors [4,34,96], batteries [54], solar cells [55], and organic field effect transistors (OFETs) [56], through SAM modification [57]. The working electrodes modified with SAM are depicted in Figure 4. The presence of an SAM on the working electrode structure acts as resistor, which contributes to the total impedance; thus, the study of SAM at a nanoscale level will help to tailor the chain length, create a defect-free electrode surface, and improve biosensor performance [97]. The layer orientation of the SAM tilts and twists at angles to the planar substrate surface, which varies with the material connected to the SAM; the presented atoms depend on the SAM composition with thickness-controlled layers [58]. A representative SAM structure consists of a head group (anchoring group), backbone (linkage), and tail group (functional group) [54,55,56]. The functionality of an SAM provides specific affinity to a substrate, while the alkanethiols on its structure enable adsorptability on the noble and coinage metals to form a high-order organic layer [98,99]. The thickness of an SAM is around 10 to 100 nm, and the deposition of an SAM on a metal surface can be performed by many techniques such as microcontact printing, scanning probes, and beams of photons, electrons, or atoms [4,34,57,58]. The SAM is deposited on a planar substrate that may be polycrystalline or single crystalline with a limited boundary [58]. Normally, Au- or Pd-based substrates are used for SAM [100,101] more frequently than other elements such as Ag-based substrates because of high conductivity, ease of bonding with the thiols group, avoidance of oxidation in the ambient environment, and nontoxicity to cells [102,103]. During SAM processing, ethanol has been used as a solvent because of its easy dissolution of alkanethiols, low cost, high purity, and low toxicity. The existence of terminally functional groups on the SAM surface allows for the immobilization of antibodies, enzymes, DNA, polypeptides, and proteins [56]. The ion–pair interaction on the SAM surface is also strongly affected by the pH range [104]. Both single and mixed SAMs can be used in micro/nano electronic devices owing to tunable SAM configurations, enhancement of stability, and modulation of the rectification properties, improving device performance [59]. To conclude, research on the biocompatible surface of SAMs facilitates electron transfer between electrodes and localized immunoreaction that contributes to the improvement of electrochemical sensors.

SAM construction on the working electrode can be carried out by diverse organic compounds, including organosulfur [58,105], organosilanes [105], phosphates [106], and carboxylic acid [105,107]. Each of them required a type of substrate corresponding to head group structure. In the organosulfur case, thanks to the sulfur-containing group, Au-S chemical bonding formation can be easily achieved, facilitating this for the head group of alkanethiols anchoring on the Au surface by reductive elimination of the hydrogen. The bonding energy of the Au-S was estimated to be roughly 40 kcal·mol^−1^ [54,56,92]. Organosulfur-compounds-based SAM in electrochemical sensors has been employed for Aβ peptides detection [4,42,53,85]. In the case of organosilanes, silanol of the organic precursor can attach to hydroxylated surfaces, for example, silicon oxide (SiO_2_), alumina, glass, zinc oxide (ZnO), indium oxide (In_2_O_3_), mica, and germanium dioxide (GeO_2_) [56,105]. Generally, hydroxyl group is not available on such kinds of surfaces; therefore, the substrates should be treated by utilizing piranha solution or oxygen plasma to obtain required surfaces [56]. Generation of the -OH group after treatment makes the substrates hydrophilic, promoting the formation of a highly ordered monolayer. Compared to organosilanes and organosulfur compounds, which have been utilized frequently in fabricating SAM, whereas an insignificant amount of carboxylic-acid- and phosphate-based SAM was also studied on metal oxide substrates [105,107]. Based on the above analyses, the selection of SAM organic precursor strongly depends on the kinds of utilized substrates. Furthermore, SAM length, SAM concentration, SAM moiety, temperature, and incubation time decide the quality of the produced SAM, which therefore directly impacts SAM impedance. What is more, the type of moieties and their distribution determine the number of immobilized antibodies or enzymes, and these accompany biochemical reaction efficiency as well as sensor performance.

SAMs have been applied in electrochemical biosensors for the detection of Aβ peptides, an Alzheimer’s biomarker. Among several abovementioned precursors for SAM formation, the application of organosulfur in manufacturing Aβ electrochemical sensor is predominant, in which pristine Au electrode has been linked with thiol-groups. Indeed, Hien et al. used an interdigitated chain-shaped electrode with an Au surface [4]: 6-mercaptohexanoic acid (MHA) was attached to the surface to form an SAM and activated with EDC/NHS to prepare for anti-Aβ antibody conjugation. The fabricated sensor enabled the detection of Aβ_1-42_ with a linear range of 10^−3^–10^3^ ng/mL and a low LOD of 100 pg/mL in HS and approximately 500 pg/mL in CSF. Liu et al. prepared an SAM by incubating the cleaned gold electrode with MPA in the dark for 12 h and then activated the SAM with EDC/NHSS to conjugate antibody by cross-linking [42]. In addition, Aβ(1-16)-heme-AuNP was generated by a ligand exchange reaction between Aβ(1-16)Cys and citrate-stabilized AuNPs. Aβ(1-16) and Aβ(1-16)-heme-AuNPs were detected by casting on the mAb-covered electrode for 10 min and using the voltametric technique in an air-saturated solution. Aβ detection was performed in a spiked sample with an Aβ(1-40)/Aβ(1-42) ratio of 6:1, similar to the real ratio in the CSF, resulting in a linear range of 0.02–1.50 nM with an LOD of 10 pM. Similarly, Dai et al. fabricated an SAM on the Au surface to conjugate antibodies for β-amyloid 42 detection. The sensor detected Aβ with a linear range of 0.0675–0.5 µg/mL [85]. Supraja et al. prepared an electrochemical immunosensor with an SAM made from MPA for detecting Aβ with a detection range of 1 fg/mL–1 ng/mL and an LOD of 0.146 fg/mL and 0.638 fg/mL in spiked buffer and plasma samples, respectively, as presented above [49]. Compared to the organosulfur-precursor-related publication, SAM formation based on organosilanes, carboxylic acid, and phosphates precursors for Aβ electrochemical sensors is very limited.

SAMs on electrochemical biosensor electrodes enable the conjugation of the antibody, which proves the immunosensor’s ability to detect the Aβ biomarker with a low LOD. Basically, SAM preparation is a type of electrode modification, and selecting the appropriate SAM with thickness-controlled layers can increase sensitivity, and therefore improve the LOD, an important factor for electrochemical biosensors in biomedical applications. All studies discussed in the present review are tabulated in Table 1.

## 4. Conclusions and Future Perspectives

This review presented electrochemical sensors for detecting a biomarker of Alzheimer’s disease with a discussion of material modification for the working electrode which exhibits promising ability in the detection of the Aβ biomarker in human serum, blood, saliva, and CSF at a low LOD of picomoles to femtomoles per liter. Especially, the use of SAMs improved the electrode surface, which is a conjunction between the antibody and pristine electrode. SAM demonstrated advantages in antibody conjugation, improved orientation, and conjunction bridge for electron transfer. However, SAM still has some drawbacks, such as the time-consuming process, difficulty in uniformly controlling SAM, and defect formation during processing.

To surmount these obstacles, research on SAM design for electrochemical sensors requires progress in the following areas:

1. Screening the organic substance for SAM formation to precisely tailor the surface properties down to the nanoscale. Relying on a specific purpose, SAM can be prepared on various substrates, e.g., silicon, Au, and metal oxide; therefore, the type of organic precursors should be chosen appropriately, then other factors, namely chain length and functional group, also should be considered for optimization.
2. Reducing time processing by avoiding the activated step without utilizing the NHS/EDC. In aforementioned works, the functional groups must have been activated by NHS/EDC to graft suitably functional groups serving for antibody immobilization. Such work consumes time and makes progression complicated. Thus, utilization of the precursor with desired functional groups can shorten the processing time.
3. Controlling the thickness of the layer by employing cutting-edge technologies. The design and fabrication of SAM on the working electrode surface can be performed by various methods, for instance, micro contact printing, scanning probe lithography, and photo-induced pattering. Each method generates its own SAM thickness, which significantly affects the electron transfer ability. Hence, for a specific organic precursor, the construction of SAM by employing the cantilever can contract SAM thickness compared to other method counterparts. However, the EFM machine is too pricey and must be operated by skilled operators.
4. The reasonable design of defect-free SAMs and study at the molecular scale to enhance sensor performance. SAM construction on the specific surface usually produces structural defects, causing a reduction in the electrochemical sensor performance. Thus, the study of SAM formation at the nanoscale can make the structural defects controllable.

## Figures and Tables

**Table 1:** Studies of Aβ detection using electrochemical biosensors.

No.	Material	Analyte	Bio-Fluids	Detection Technique	Linear Range	LOD	Ref.
1	Au/SAM/NHS-EDC/Ab-Aβ42	Aβ(1-42)	Undiluted HR ^(^*^)^	DPV	0.0675–0.5 g/mL	NA ^(^**^)^	[78]
2	-DEP/Au/MHDA-EDC/Ab-Aβ -DEP/Au/MHDA-EDC/protein G/Ab-Aβ	Aβ(1-40) Aβ(1-40)	PBS PBS	EIS EIS	1 to 10^3^ nM 10 to 10^5^ pM	2.65 nM 0.57 nM	[55]
3	CFP/AuPt/DNA aptamer	AβO	HS	DPV	0.5 to 10^4^ pg/mL	0.16 pg/mL	[56]
4	β-CD modified Au electrode/MCH/Ad-Pr(95-110)/Ag	AβOs	PBS	LSV	20 to 10^5^ pM	8 pM	[75]
5	GCE/SNF/(EDC-NHS)/Aβ42 antibodies/BSA	Aβ(1-42) Aβ(1-42)	Spiked sample Plasma	EIS	1 to 10^7^ fg/mL 1 to 10^6^ fg/mL	0.146 fg/mL 0.638 fg/mL	[49]
6	rGO/Pyr-NHS/H31L21/Ab-Aβ/BSA	Aβ(1-42)	Human blood	DPV	11 to 55.10^3^ pM	2.398 pM	[57]
7	NiFe_2_O_4_ decorated GO/Au/Ab-Aβ/BSA	Aβ(1-42)	PBS	DPV	1 to 10^3^ mg/mL	3.0 pg/mL	[65]
8	G/Co_9_S_8_-Pd/Ab-Aβ/BSA	Aβ	Spiked CSF	Amperometry	0.1 to 50.10^3^ pg/mL	41.4 fg/mL	[64]
9	Au-VG/CC/PrP^c^/Aptamer-poly T-CuNPs	Aβ oligomer	PB	DPV	10 to 2200 pM	3.5 pM	[62]
10	SPGE/pDAN/Ab-Aβ/BSA	Aβ(1-42)	Spiked plasma	DPV	1 to 1000 pg/mL	1.4 pg/mL	[63]
11	PrP^c^/AuNPs-E-PTAA PrP^c^/AuNPs-E-Ppy-2-COOH PrP^c^/AuNPs-E-Ppy-3-COOH	AβO	CSF	EIS	10^−9^ to 10^3^ nM 10^−9^ to 10^3^ nM 10^−9^ to 10^3^ nM	NA NA 10^−9^ nM	[88]
12	BSA/CGG CGG Chit/CCG DA/CCG DNA/CCG Gel/CCG PEG/CCG	Aβ40 Aβ42	CSF	EIS	NA	NA	[87]

^(^*^)^ HR: human serum. ^(^**^)^ NA: not available.

## Data Availability

Not applicable.
